# Regulation of Inflammatory Response and the Production of Reactive Oxygen Species by a Functional Cooked Ham Reformulated with Natural Antioxidants in a Macrophage Immunity Model

**DOI:** 10.3390/antiox8080286

**Published:** 2019-08-06

**Authors:** Antonio Serrano, Gaspar Ros, Gema Nieto

**Affiliations:** Department of Food Technology, Nutrition and Food Science, Veterinary Faculty, University of Murcia, Espinardo Campus, Espinardo, 30100 Murcia, Spain

**Keywords:** functional meat, functional food, cooked ham, polyphenol, antioxidant, reactive oxygen species, anti-inflammatory, cytokines

## Abstract

Nowadays, more consumers demand healthier products. A way to offer such products is to functionalize them using health-promoting bioactive compounds. Meat and meat products are high in essential nutrients; however, their excessive consumption implies a high intake of other substances that, at levels above recommended uptake limits, have been linked to certain non-communicable chronic diseases. An effective way to reduce this danger is to reformulate meat products. In this study, natural botanical extracts rich in anti-inflammatory and antioxidant compounds were used to improve the health properties of a cooked ham with an optimal nutritional profile (i.e., low in fat and salt). The RAW 264.7 mouse cell line was used as an inflammatory model and was stimulated with *Escherichia coli* lipopolysaccharide to evaluate changes in inflammatory biomarkers such as tumour necrosis factor alpha, the interleukins (ILs) IL-1β and IL-6, nitric oxide and intracellular reactive oxygen species (ROS). The results showed that the use of natural extracts in optimized cooked ham significantly downregulated inflammatory markers and reduced the levels of intracellular ROS. Thus, the present study proposed a new functional cooked ham with potential health properties via anti-inflammatory and antioxidant in vitro activity.

## 1. Introduction

Meat is an important source of essential nutrients. It provides high-quality proteins, vitamins such as B12 and B6 and minerals such as iron and selenium. However, recent studies have linked meat—mostly red and processed meats—with the incidence of non-communicable diseases such as colorectal cancer [1,2].

Although epidemiological studies support this premise, the mechanistic associations are not well understood. Meat contains several molecules that could be involved in non-communicable diseases [3]. For example, the consumption of one component of meat, heme iron—an essential source of dietary iron and a nutrient—should be moderated, as, if it is consumed in excess, it increases the risk of type II diabetes by causing oxidative damage to pancreatic β cells [4] and also causes inflammatory disruption due to the catalysis of fat peroxidation, which can lead to cardiovascular diseases [5]. On the other hand, the high density of nutrients in meat, such as fats, compel its consumption to be moderated in order to prevent metabolic syndrome [6] or diseases related to the excessive intake of saturated fats and the content of sodium in processed meats. Studies have linked pathogenesis with substances such as N-nitrous compounds [7] and heterocyclic amines [8]; however, these works have limitations, as their authors did not specify key elements such as the characteristics of thermal treatment and other processes applied to meat, which influence the development of N-nitrous compounds and heterocyclic amines [9].

During inflammatory processes, the phagocytic activation of macrophages, monocytes, and polymorphonuclear leukocytes results in the pouring of free radicals into biological tissues and an inflammatory response called respiratory burst [10]. The deregulation of those processes due to the low-grade inflammatory response implying postprandial processes [11] produces pro-inflammatory molecules and cytokines, which are linked with tumour progression [12] together with free radicals. Cytokines also have other physiological functions, such as mediating macrophage chemotaxis and angiogenesis by interleukin (IL)-1 [13]; they are also involved in the differentiation of immune cells via IL-6 [14] and have been widely used as an inflammatory biomarker [11,15,16,17]. 

In this study, in order to prevent oxidative and inflammatory deregulation, some natural compounds were selected based on previous studies of antioxidant and anti-inflammatory potential. For example, catechins have been shown to upregulate endogenous antioxidants such as superoxide dismutase and catalase [18] and to have anti-proliferative and anti-angiogenesis activities [19]. Additionally, rosmarinic acid has been demonstrated to have anti-inflammatory properties [20,21], and hydroxytyrosol, an emerging and potent antioxidant derived from *Olea europaea*, has been shown to protect against oxidative stress and inflammation [22]. Moreover, chlorogenic acids have been shown to modulate inflammation [23].

In this study, our hypotheses were (1) that processed meat plays a role in oxidative and inflammatory processes related to immunity signalling and (2) that such meat can be made healthier with the addition of natural extracts with antioxidant and anti-inflammatory properties, since a decrease in the level of pathogenesis-related biomarkers such as cytokines or reactive oxygen species (ROS) could improve the healthiness of cooked ham. To test these hypotheses, a macrophage model of inflammation was used to test both the anti-inflammatory and antioxidant potential of standard and enriched cooked ham. For this purpose, a cooked ham was selected as a base product due to its improved macronutrient profile (i.e., low sodium content and saturated fat). Then, the ham was enriched with natural extracts that can palliate the possible pathogenic effects of the meat due to the antioxidant and anti-inflammatory processes [24].

The aim of the present study was to evaluate the inflammatory role of processed meat and to develop a functional meat product enriched in natural extracts with antioxidant and anti-inflammatory properties.

## 2. Materials and Methods

### 2.1. Reagents

The enzymes pepsin (catalogue no. P7000), pancreatin (catalogue no. P1750) and α-amylase (catalogue no. A6380) for in vitro digestion, 2,4,6-tris(2-pyridil)-s-triazine (TPTZ) (catalogue no. 93285), iron (III) chloride hexahydrate (catalogue no. 31232), Folin–Ciocalteu phenol reagent (catalogue no. F9252), gallic acid (catalogue no. 91215), lipopolysaccharide from *Escherichia coli* serotype O127:B8 4.5 g/L (catalogue no. L4516), 2’,7’-dichlorofluorescin diacetate (catalogue no. D6883), Griess reagent (catalogue no. 03553) and thiazolyl blue tetrazolium bromide (catalogue no. M5655) were purchased from Sigma–Aldrich Co. (St. Louis, MO, USA). Dulbecco’s Modified Eagle Medium (4.5 g/L glucose, 3.7 g/L sodium bicarbonate, 2 mM glutamine, 1% non-essential amino acids, 1% penicillin–streptomycin) was purchased from Gibco BTL Life Technologies (Paisley, Scotland). Dimethyl sulfoxide (DMSO) and MCYTOMAG-70K plate for Luminex assay were supplied by Merck KGaA (Darmstadt, Germany).

### 2.2. Samples

BienStar^®^ ham (produced by ElPozo Alimentación, S.A., Alhama de Murcia, Murcia, Spain) is a cooked ham with an improved nutritional profile (25% less fat and 35% less sodium than a standard cooked ham). In this study, uncooked BienStar^®^ ham dough (which contains 85% pork ham, water, potassium chloride, corn dextrose, sugar, corn syrup, flavouring, sodium citrate, and sodium erythorbate) was used as the base product to develop a new functional meat. The original BienStar^®^ dough was used as the control sample (C), and two new functional meat foods were obtained by adding to one sample of dough a natural antioxidant aqueous solution containing 12.5 mg/mL of chlorogenic acid, 10 mg/mL of catechins and 1.5 mg/mL of rosmarinic acid (called CCR), and adding to another sample of dough a natural antioxidant aqueous solution containing 1.5 mg/mL of hydroxytyrosol (called CCH). Both aqueous solutions were mixed with a water standard to a concentration of 5% *w*/*w* with BienStar^®^ ham dough. The three meat samples (C, CCR and CCH) were cooked at 80 °C for 40 min.

In order to simulate physiological digestion, the cooked ham samples were digested following the method described by Minekus et al. [25]. The resulting homogenates were filtered using 0.22 µm PVDF filters in order to sterilize them for cell culturing (see Section 2.5), thus obtaining the final samples. 

### 2.3. Ferric-Reducing Antioxidant Power (FRAP) Assay

The antioxidant power of the digested samples was measured using the ferric-reducing antioxidant power (FRAP) method [26]. Subsequently, the samples were added to a cell culture in order to correlate the increased antioxidant content of the cooked ham with the modulation of oxidative status in macrophages.

To prepare the working solution, a 10 mM TPTZ solution was prepared in 40 mM HCl and then mixed with 20 mM FeCl_3_·6H_2_O in distilled water and 0.3 M sodium acetate anhydrous buffer solution at a ratio of 1:1:10 (*v*:*v*:*v*). Standard Trolox solution was prepared to determine the calibration curve at a concentration range of 10–200 μM. Samples and the standard were mixed with the FRAP working solution at 1:10 and then incubated at 37 °C for four minutes. Then, the absorbances of the samples and the standard were measured at a wavelength of 595 nm. The results were expressed as µm Trolox equivalents per 100 g of digested sample.

### 2.4. Determination of Total Phenolic Content

The total phenolic content (TPC) was determined using the Folin–Ciocalteu method [27]. A total of 100 µL of digestion liquid extracts were diluted in 3 mL distilled water and 0.5 mL of Folin–Ciocalteu reagent was added and incubated for 3 min. Then, a mixture of water and 20% sodium carbonate was added and mixed and the mixture was incubated for 20 min. Then, the absorbance of the mixture was measured at 750 nm. A standard gallic acid solution was prepared for the determination of the calibration curve.

### 2.5. Cell Culture

The RAW 264.7 macrophages were obtained from the European Collection of Cell Cultures (ECACC; Salisbury, UK; number 86010202). Cells were maintained as indicated by the provider in Dulbecco’s Modified Eagle’s Medium (4.5 g/L glucose, 3.7 g/L sodium bicarbonate) supplemented with 10% heat-inactivated fetal bovine serum and incubated at 37 °C with 5% CO_2_, 95% air atmosphere, and 95% relative humidity. The medium was replaced every two days and subcultures were made at a ratio of 1:5 in a 75 cm^2^ culture flask.

#### 2.5.1. Cell Viability

Cell viability was evaluated using 3-(4,5-dimethylthiazol-2-yl)-2,5-diphenyltetrazolium bromide assay (MTT) [28]. Cells were seeded at 2 × 10^4^ in 96 well plates at digestion extract concentrations of between 1 and 10% *v*/*v* with medium applied for 24 h. Then, after removing the medium, 100 µL of the MTT solution (5 mg/mL in phosphate-buffered saline) was added and the mixture was incubated for 4 h at 37 °C. A total of 100 µL of DMSO was added to each well to dissolve formazan and then the absorbance was measured at 540 nm. Results lower than 90% of cell viability resulted in the elimination of the sample.

#### 2.5.2. Lipopolysaccharide-Induced Inflammation Assay

Lipopolysaccharide (LPS) from *E. coli* was used to stimulate RAW 264.7 to produce free radicals, NO, and IL [29]. Cells were seeded at a density of 1 × 10^4^ and incubated for 24 h to ensure total adherence and functionality. Standard media were enriched with 5% *v*/*v* of digested distilled water as a control without sample for measuring the basal status without inflammation and total stimulus with LPS. The same procedure was performed for measuring changes with control digested cooked ham and the CCH and CCR digested enriched cooked hams. Then, 200 µL of each enriched medium was added to the cell cultures and the mixtures were left for 24 h. After the incubation, the enriched medium was removed and 200 µL of medium with 1 µg/mL LPS was added to all wells except the basal control and the mixture was left for 24 h. After the last incubation, the medium was stored for NO and interleukin measurement and fresh medium was added in preparation for the ROS assay.

#### 2.5.3. Measurement of Reactive Oxygen Species

The 2′-7′-dichlorofluorescein diacetate (DCFH-DA) was used to measure ROS [30]. Cell supernatant was enriched with 12.5 µM DCFH-DA and incubated for 30 min at 37 °C. After incubation, the supernatant was removed, plates were washed twice with phosphate buffered saline (pH = 7.4) and fluorescence was measured at *λ_ex_* = 488 nm and *λ_em_* = 530 nm.

#### 2.5.4. Measurement of Nitric Oxide

Cell supernatant for NO determination was mixed 1:1 with Griess reagent and incubated at room temperature for 20 min. Then, absorbance was measured at a wavelength of 540 nm.

#### 2.5.5. Measurement of Cytokines

The interleukins IL-1β and IL-6 and tumour necrosis factor alpha (TNFα) were measured using Luminex multiplex immunoassay technology. A Luminex MAGPIX system with a MCYTOMAG-70K plate was used. As per the manufacturer’s instructions, a total of 25 μL of LPS-stimulated RAW 264.7 supernatant was used to determine cytokine concentrations using the Luminex xPONENT software. The results were obtained in pg/mL and expressed as a percentage of respective cytokine inhibition.

#### 2.5.6. Statistics

Statistical analysis was performed using the GraphPad Prism 7.0 software for Windows (GraphPad Software, San Diego, CA, USA). Results were expressed as the mean plus or minus of the standard error of the mean (SEM). Multiple comparisons were performed using one-way (FRAP, TPC, ROS and NO results) or two-way ANOVA (cytokine results) followed by Tukey’s multiple comparison test. The *p*-values less than 0.0001 were considered significant. 

## 3. Results

### 3.1. Preliminary Antioxidant Assays

**Figure 1:**
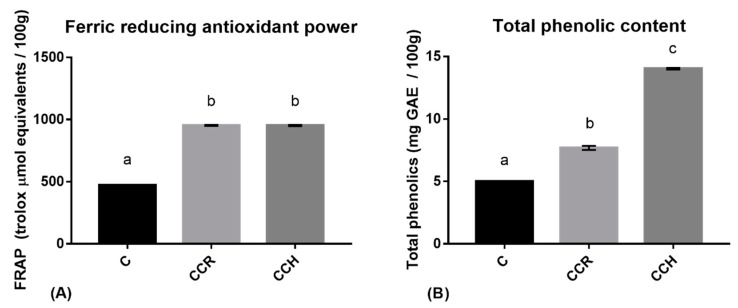
The results of the ferric-reducing antioxidant power (FRAP) assay (**A**) and total phenolic content (**B**) for the control cooked ham (C), the cooked ham reformulated with 5% *w*/*w* of an aqueous solution of chlorogenic acids, catechins, and rosmarinic acid (CCR), and the cooked ham reformulated with 5% *w*/*w* of an aqueous solution of chlorogenic acids, catechins, and hydroxytyrosol aqueous solution (CCH). Different letters represent differences that are statistically significant (*p* < 0.0001).

The results of the FRAP assay, which was performed in order to check for increases in antioxidant activity, showed that both of the enriched cooked hams presented statistically significant differences (*p* < 0.0001), with the antioxidant capacity of each ham being twice that of the control ham (Figure 1).

The phenolic content of the CCR ham was 54.21% higher than that of the control ham, while that of the CCH ham was 180% higher than that of the control ham.

These results verify the effectiveness of reformulating cooked ham in terms of antioxidant capacity and phenolic content. The differences in the phenolic content and antioxidant capacity of the CCH and CCR hams indicate that a lower phenolic content can be associated with the same reducing activity depending on the compound added (hydroxytyrosol or rosmarinic acid, respectively).

### 3.2. Intracellular Reactive Oxygen Species

The downregulation of ROS production compared to non-treated stimulated RAW 264.7 is expressed as % of ROS inhibition. This parameter decreased significantly when macrophages were treated with the digested extract from the control cooked ham, with an inhibition of 19.28 ± 0.31%. This shows that, even without reformulating, the cooked ham had intracellular antioxidant properties. Technological antioxidants for food preservation could have protective intracellular effects against ROS.

**Figure 2:**
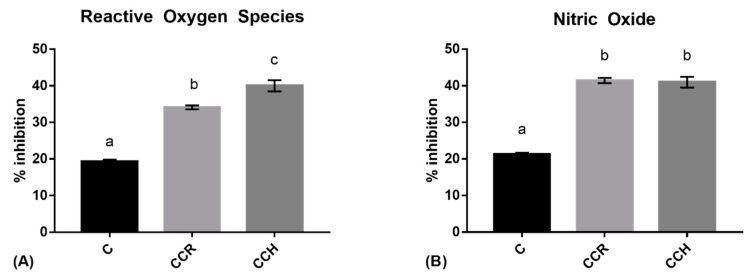
The ability of digested extracts of ham to inhibit reactive oxygen species (ROS) (**A**) and to inhibit nitric oxide production (**B**) from lipopolysaccharide-activated RAW 264.7 macrophages. Different letters represent statistically significant differences (*p* < 0.0001).

As shown in Figure 2A, when the cooked ham was reformulated with CCR or CCH prior to digesting and treating macrophages, its ability to decrease the level of intracellular ROS increased significantly, with higher ROS inhibition being observed with the CCH formula than the CCR formula. The differences among the two formulas can be attributed to the presence of hydroxytyrosol, which seems to be significantly more effective at inhibiting ROS than rosmarinic acid.

The fact that both the reformulated hams showed a higher inhibition of ROS and NO production (*p* < 0.0001) than the control ham indicates an improvement in the reduction of intracellular ROS and NO production.

### 3.3. Nitric Oxide

As shown in Figure 2B, the amount of extracellular NO was slightly lower for the cells treated with the digested extract of the standard cooked ham compared to non-treated stimulated RAW 264.7. As was observed for ROS, reformulating with natural extracts significantly increased NO inhibition in stimulated macrophages (*p* < 0.0001).

### 3.4. Cytokine Measurement

The release of cytokines, which is used as a biomarker of inflammation, was not significantly different among polyphenol-enriched cooked hams, despite the fact that higher TNFα inhibition was observed in the CCH enriched ham. However, the hams treated with CCR and CCH, respectively, inhibited the extracellular signalling of IL-1β and IL-6. Additionally, the ham treated with CCH more effectively inhibited TNFα.

**Figure 3:**
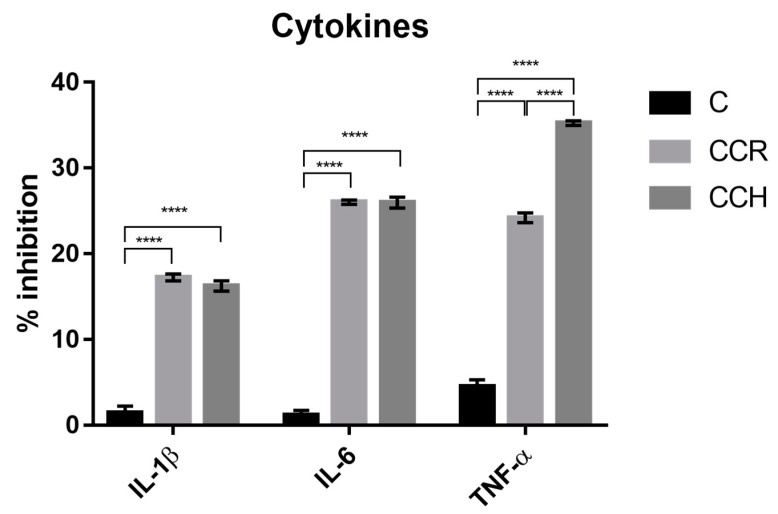
The effect of digested extracts of the three cooked hams on the inhibition of the cytokines interleukin (IL)-1β and IL-6 and tumour necrosis factor alpha (TNFα) in lipopolysaccharide-activated RAW 264.7 macrophages. **** *p* < 0.0001.

The results in Figure 3 show the effectiveness of polyphenol in modulating the inflammatory macrophage response, which could indicate a new strategy to produce healthier meat products.

Although the control ham showed slight inhibition of IL-1β, IL-6, and TNFα, the inhibition of these cytokines was significantly higher for the hams reformulated with polyphenol blends (*p* < 0.0001).

## 4. Discussion

Due to the widespread consumption of meat, there has been widespread research into the production of healthier meat products. Some authors have focused on inhibiting potential carcinogenic substances generated during meat processing using natural antioxidants [31,32]—such as rosemary extract, which is rich in rosmarinic acid—which have been shown to decrease lipid oxidation [33,34,35] and improve the healthiness of processed meat.

Previous studies on the antioxidant capacity of meat have shown that, by itself, meat acts as a radical scavenger or reducing agent [36] with an activity similar to that measured in non-reformulated meat in this assay. Since pigs are monogastric animals, the antioxidant capacity of their meat can be influenced by their diet [37]. Such an influence may explain the polyphenol content of the control sample in this study; however, it does not affect the comparison of the results since the same meat was used to prepare the three samples. Previous studies showed that, in a standard diet, meat and meat products represent up to 10.51% of total antioxidant intake [38]. However, in this study, the total phenolic content of the meat product was found to increase after treatment with natural extracts, doubling the antioxidant capacity of the meat product. 

These findings are consistent with the fact that the control cooked ham was demonstrated to have antioxidant properties. The denaturation of proteins through heat processing has been shown to release antioxidant peptides and some Maillard reaction products [36]. This can explain the antioxidant properties and the presence of technological antioxidants in processed meats such as the cooked ham used in the present study.

The level of intracellular ROS can be reduced both by enhancing endogenous intracellular antioxidants using polyphenols [39] and the antioxidant capacity of technological antioxidants evaluated by the FRAP method. The differences observed in this study between the CCH and CCR hams can be explained by the fact that higher values of TPC were observed in the CCH ham and that the hydroxytyrosol used to treat this ham was highly bioavailable [40] compared to other polyphenols [41], and was, therefore, less degraded by the digestion process used in the present study.

Antioxidants have been widely described as protective agents against degenerative diseases [42] produced by physiological mechanisms which involve the pouring of ROS [43]. The inhibition of these radicals by untreated and reformulated cooked ham may, therefore, lead to the prevention of oxidative damage. However, more detailed in vivo studies should be performed.

Nitric oxide is synthesized by macrophages as a signalling molecule of inflammation and a toxic defence against infectious organisms [44]. Its presence indicates the activation of NO synthase, which in this study was due to the simulated infectious stimulus by an *E. coli* lipopolysaccharide. Reducing postprandial physiological inflammation could prevent cardiovascular diseases caused by lipemia enteric processes [45] and some pathways of colon cancer promotion [44]. A previous study observed inhibitions of NO emissions (measured as nitrite) of around 50% by treating cell cultures with pure concentrations of flavonoids such as quercetin and kaempferol at a concentration of 50 µM [46]. Furthermore, another study related heme oxygenase-1 protein expression with the inhibitory effects of flavonoids on NO emission [47].

In our research, the control cooked ham showed a slight inhibition of NO production from macrophages. In macrophages, NO is synthesized by NO synthase, whereas superoxide is mainly produced by nicotinamide adenine dinucleotide phosphate oxidase. Peroxynitrite is produced in vivo when reactions occur between superoxide and NO. Considering that peroxynitrite generation has been attributed to inflammatory diseases such as chronic heart failure, myocardial infarction, stroke, diabetes, and cancer, the consumption of cooked ham (under a balanced diet) may control these inflammatory diseases.

Heat processes and digestion can release peptides that modulate nitric oxide in a similar way to that achieved by Song et al. [48], who used peptides from the sea cucumber. In the present study, reformulating ham with CCH and CCR was found to significantly increase the capacity to inhibit the production of ROS and NO compared to the control cooked ham, thus increasing the anti-inflammatory capacity of the ham.

Cytokines have been shown to be inflammatory mediators [49]. The results of the present study show that the control cooked ham slightly inhibited the production of TNFα by stimulated macrophages, while the CCH and CCR reformulated hams both significantly downregulated the secretion of IL-1β, IL-6, and TNFα. The cytokine IL-6 has been described as both inflammatory and anti-inflammatory; under specific conditions, including LPS-induced inflammation, it was considered to be inflammatory [50]. Reducing the level of inflammatory cytokines could allow the prevention of inflammatory diseases, as suggested by Arranz et al. [51], using rosemary extract, and by Wu et al. [52], using dioscin. Other foods have been tested to determine their influence on inflammatory status. For example, digested einkorn-based bread was tested with stimulated Caco-2 cells, achieving a significant reduction in IL-6 production [53], and similar levels of ROS and NO inhibition were observed using digested bread with HepG2 cells [54]. Cytokines were also shown to be downregulated by bile salt used in digestion [55]. In the present study, positive and negative inflammation control wells were treated with digested distilled water at the same concentration as in the sample wells.

## 5. Conclusions

The present study found that cooked ham with an optimal nutritional profile had antioxidant and anti-inflammatory properties related to the inhibition of substances which are involved in gut inflammation—namely, reactive oxygen species, nitric oxide, and cytokines—produced by macrophages. These health-promoting inhibitory properties were significantly increased when the cooked ham was enriched with bioactive phytochemicals. In conclusion, the incorporation of botanical extracts rich in phenolic compounds (i.e., chlorogenic acid, catechins, rosmarinic acid, and hydroxytyrosol) in cooked ham is an excellent strategy to produce a healthy functional meat product.
